# Side Effects of mRNA-Based COVID-19 Vaccine: Nationwide Phase IV Study among Healthcare Workers in Slovakia

**DOI:** 10.3390/ph14090873

**Published:** 2021-08-29

**Authors:** Abanoub Riad, Barbora Hocková, Lucia Kantorová, Rastislav Slávik, Lucia Spurná, Adam Stebel, Michal Havriľak, Miloslav Klugar

**Affiliations:** 1 Czech National Centre for Evidence-Based Healthcare and Knowledge Translation (Cochrane Czech Republic, Czech EBHC: JBI Centre of Excellence, Masaryk University GRADE Centre), Institute of Biostatistics and Analyses, Faculty of Medicine, Masaryk University, 625 00 Brno, Czech Republic; lucia.kantorova@mail.muni.cz (L.K.); klugar@med.muni.cz (M.K.); 2 Department of Public Health, Faculty of Medicine, Masaryk University, 625 00 Brno, Czech Republic; 3 Department of Maxillofacial Surgery, F. D. Roosevelt University Hospital, 975 17 Banska Bystrica, Slovakia; bhockova@nspbb.sk (B.H.); rslavik@nspbb.sk (R.S.); astebel@nspbb.sk (A.S.); 4 Department of Prosthetic Dentistry, Faculty of Medicine and Dentistry, Palacky University, 775 15 Olomouc, Czech Republic; 5 Department of Anesthesiology, F. D. Roosevelt University Hospital, 975 17 Banska Bystrica, Slovakia; 11lucia@pobox.sk; 6 Clinic of Otorhinolaryngology and Head and Neck Surgery, Central Military Hospital and Faculty Hospital, 034 26 Ružomberok, Slovakia; michal.havrilak@gmail.com; 7 Institute of Health Information and Statistics of the Czech Republic, Palackého náměstí 4, 128 01 Prague, Czech Republic

**Keywords:** BNT162b2 vaccine, cross-sectional studies, COVID-19, drug-related side effects and adverse reactions, health personnel, mass vaccination, prevalence

## Abstract

mRNA-based COVID-19 vaccines such as BNT162b2 have recently been a target of anti-vaccination campaigns due to their novelty in the healthcare industry; nevertheless, these vaccines have exhibited excellent results in terms of efficacy and safety. As a consequence, they acquired the first approvals from drug regulators and were deployed at a large scale among priority groups, including healthcare workers. This phase IV study was designed as a nationwide cross-sectional survey to evaluate the post-vaccination side effects among healthcare workers in Slovakia. The study used a validated self-administered questionnaire that inquired about participants’ demographic information, medical anamneses, COVID-19-related anamnesis, and local, systemic, oral, and skin-related side effects following receiving the BNT162b2 vaccine. A total of 522 participants were included in this study, of whom 77% were females, 55.7% were aged between 31 and 54 years, and 41.6% were from Banska Bystrica. Most of the participants (91.6%) reported at least one side effect. Injection site pain (85.2%) was the most common local side effect, while fatigue (54.2%), headache (34.3%), muscle pain (28.4%), and chills (26.4%) were the most common systemic side effects. The reported side effects were of a mild nature (99.6%) that did not require medical attention and a short duration, as most of them (90.4%) were resolved within three days. Females and young adults were more likely to report post-vaccination side effects; such a finding is also consistent with what was previously reported by other phase IV studies worldwide. The role of chronic illnesses and medical treatments in post-vaccination side effect incidence and intensity requires further robust investigation among large population groups.

## 1. Introduction

On 21 December 2020, the European Medicines Agency (EMA) recommended the first COVID-19 vaccine, Comirnaty, for conditional marketing authorisation across the European Union (EU) [1]. This approval was depicted as a landmark in our journey towards controlling the coronavirus disease (COVID-19) pandemic caused by severe acute respiratory syndrome coronavirus 2 (SARS-CoV-2), first described in Wuhan, China [2]. Comirnaty is a messenger ribonucleic acid (mRNA-based) COVID-19 vaccine developed and manufactured by BioNTech Manufacturing GmbH (Mainz, Germany) and Pfizer Inc (New York, NY, USA); therefore, its well-known market name is Pfizer-BioNTech COVID-19 vaccine, and it is scientifically referred to as BNT162b2 [3]. Comirnaty requires two intramuscular doses three to six weeks apart to achieve optimal immunization [3]. Two weeks later, the EMA approved another mRNA-based COVID-19 vaccine, Spikevax [4]. Spikevax vaccine is developed by Moderna Inc (Cambridge, MA, USA) and manufactured and distributed in the EU by Moderna Biotech Spain S.L. (Madrid, Spain); therefore, its market name is Moderna COVID-19 vaccine, and the scientific name is mRNA-1273 [5]. Similar to Comirnaty, Spikevax requires two intramuscular doses four weeks apart to achieve optimal effectiveness.

On 26 December 2020, the COVID-19 vaccine rollout started in Slovakia following a prioritisation strategy stipulated by the Slovak Ministry of Health (MoH) decree [6]. According to the decree, vaccination of the Slovak population would be realised within eight consecutive phases [7]. In “Phase I”, frontline healthcare workers, medical and healthcare students, and essential public workers were planned to be inoculated; therefore, most of their vaccination relied on the available vaccines at that time, which were mRNA-based vaccines [7].

To date, the globally available COVID-19 vaccines belong to one of the following technologies: (a) mRNA-based, (b) viral vector-based, (c) protein subunit, and (d) whole virus vaccines. The mRNA-based technology is the youngest technology among all other vaccine manufacturing technologies; therefore, it was a target of anti-vaccine campaigns due to a lack of prior experience of using it in mass vaccination [8]. The mRNA-based vaccines use mRNA molecular templates to deliver the genetic information to build the spike protein antigen, instead of delivering the antigen itself, to trigger the targeted immune response and production of SARS-CoV-2 antibodies [9].

COVID-19 vaccines, similar to all other novel pharmaceutical products, are subjected to post-marketing evaluation by drug regulators and collaborating academic and clinical institutions. Pharmacovigilance systems can be broadly divided into three main types: (a) passive surveillance, (b) active surveillance, and (c) hybrid surveillance systems [10]. Passive surveillance systems rely entirely on the spontaneous reporting of post-vaccination side effects by healthcare professionals or sometimes by the individuals or their guardians, while active surveillance systems follow a phase III-like approach where they tend to perform epidemiologic studies for the vaccinated individuals to collect their self-reported side effects [11,12].

The Medicines and Healthcare products Regulatory Agency (MHRA) of the United Kingdom (UK) was one of the first in the world to adopt a multi-layered system for COVID-19 vaccines vigilance that incorporated the following elements: (a) an enhanced passive surveillance, (b) rapid cycle and ecological analyses, (c) targeted active monitoring through the MHRA native system “Yellow Cards”, and (d) formal epidemiologic studies in collaboration with independent institutions, e.g., the London School of Hygiene & Tropical Medicine (LSHTM) [12]. These hybrid systems are needed now more than ever because they can overcome the drawbacks of sole passive surveillance and active surveillance systems. Jęśkowiak et al. (2021) evaluated the Polish passive surveillance system and concluded that it was unreliable in the case of COVID-19 vaccines vigilance, as they compared its data to targeted active studies and found serious discrepancies [13].

As of 21 July 2021, the Slovak State Institute for Drug Control (SIDC) received 7651 reports of possible side effects related to COVID-19 vaccines, of which 702 (9.3%) were serious [14]. Out of the 2,810,050 administered doses of BNT162b2, 0.123% reported side effects, and 0.013% reported serious side effects. Compared to that, out of the 423,090 doses of mRNA-1273, 0.224% reported side effects, and 0.014% reported serious side effects [14].

Aversion to post-vaccination side effects was described by the Strategic Advisory Group of Experts on Immunization (SAGE) of the World Health Organization (WHO) as a key promoter for vaccine hesitancy among different population groups [15]. Not only can the life-threatening side effects trigger vaccine hesitancy, but the minor side effects that are usually self-limiting can also prevent individuals from making the decision to receive a vaccine, as even these minor side effects can lead to interruption of daily routine and absence from work/school [16]. Therefore, one of the effective approaches to counter vaccine hesitancy is to continuously and transparently monitor the safety of vaccines to enhance public confidence [17,18].

The overarching aim of this study was to evaluate the self-reported side effects of the mRNA-based COVID-19 vaccine, BNT162b2, among healthcare workers in Slovakia. The primary objective was to estimate the prevalence of each local, systemic, oral, and skin-related side effect and to estimate the duration and intensity of the solicited side effects. The secondary objective was to evaluate the association of post-vaccination side effects and demographic and medical variables that could act as potential risk factors for side effect incidence and intensity.

## 2. Results

### 2.1. Demographic Characteristics

Out of the 536 Slovak healthcare workers who filled in the questionnaire completely, five respondents received viral vector-based vaccines, mainly AstraZeneca-Oxford COVD-19 vaccine, and nine respondents received either mRNA-1273 or only the first dose of BNT162b2.

A total of 522 participants were included in the downstream analyses: 402 (77%) were females, and 120 (23%) were males. The mean age of the participants was 37.77 ± 11.61 years, with 34.3% being young adults (18–30 years old), 55.7% being middle-aged adults (31–54 years old), and 10% being old adults (≥55 years old) (Table 1).

**Figure 1:**
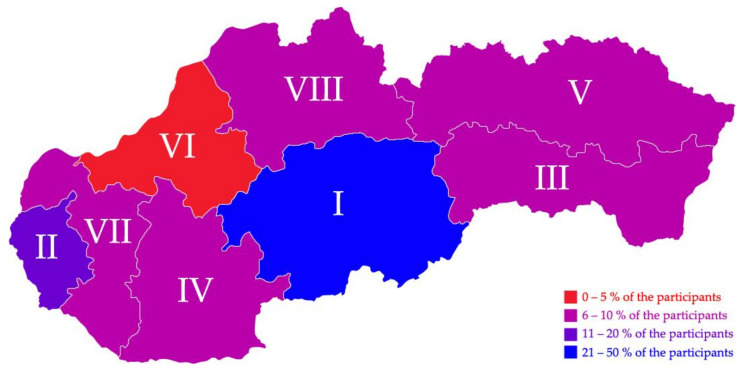
Geographic distribution of participating Slovak healthcare workers, February–March 2021 (*n* = 522).

The most common professions were physicians (46.9%), followed by nurses (18.6%) and dentists (12.3%). The majority (36.4%) of the participants had 1–5 years of work experience. The most contributing region was Banská Bystrica (41.6%) followed by Bratislava (19.5%) and Košice (7.3%) (Figure 1).

### 2.2. Medical Anamnesis

About one-quarter (25.9%) of the participants reported having at least one chronic illness without a statistically significant (*χ*^2^ = 0.919; *Sig*. = 0.338) difference between females (26.9%) and males (22.5%). The young adults significantly had (*χ*^2^ = 27.384; *Sig*. < 0.001) the lowest prevalence of chronic illnesses (16.2%), followed by middle-aged (27.1%) and old adults (51.9%).

The most common chronic illness was chronic hypertension (6.9%), followed by thyroid disease (6.5%), bowel disease (3.3%), and allergies (2.3%). Only thyroid disease was significantly (*χ*^2^ = 8.256; *Sig*. = 0.004) different between females (8.2%) and males (0.8%). On comparing the young adults vs. middle-aged adults vs. old adults, cardiac disease (0% vs. 1.7% vs. 5.8%), chronic hypertension (1.1% vs. 5.2% vs. 36.5%), diabetes mellitus (0% vs. 0.7% vs. 9.6%), and rheumatoid arthritis (0% vs. 0.7% vs. 3.8%) had a significant growing pattern with age, thus indicating the role of ageing in developing these chronic diseases among healthcare workers in Slovakia.

Less than one-third (31.8%) of the participants were taking at least one medication regularly with a statistically significant (*χ*^2^ = 5.151; *Sig*. = 0.023) difference between females (34.3%) and males (23.3%). The young adults significantly had (*χ*^2^ = 29.574; *Sig*. < 0.001) the lowest level of regular medication consumption (21.8%), followed by middle-aged (32.6%) and old adults (61.5%).

The most common medication was antihypertensive drugs (9.6%), followed by thyroid hormones (7.7%), antihistamines (7.7%), immunosuppressive drugs (3.6%), and nonsteroidal anti-inflammatory drugs (2.9%). Both thyroid hormones (*χ*^2^ = 10.272; *Sig*. = 0.001) and the drugs for gastroesophageal reflux disease (2-S Fisher’s Exact Test; *Sig*. = 0.027) were significantly different among females (9.7% and 0.5%) and males (0.8% and 3.3%), respectively. The consumption of drugs was significantly (*χ*^2^ = 29.574; *Sig*. < 0.001) more common among old adults (61.5%) than middle-aged (32.6%) and young adults (21.8%). In contrast to this age-dependent pattern, contraceptives and the drugs for gastroesophageal reflux disease were more common among young adults (1.7% and 1.7%) than middle-aged (0.7% and 1%) and old adults (0% and 0%), respectively (Table 2).

### 2.3. COVID-19-Related Anamnesis

The vast majority of the participants did not have prior COVID-19 infection (86.8%), and there was no significant (*χ*^2^ = 5.151; *Sig*. = 0.966) difference between females (13.2%) and males (13.3%) in terms of prior infection.

While 71.7% of the male participants had been in direct contact with confirmed COVID-19 cases, only 57.7% of the female participants reported so, and the gender-based differences were statistically significant (*χ*^2^ = 7.560; *Sig*. = 0.006).

The mean duration between the second dose and the first dose was 28.46 ± 6.52 days, and the males (29.55 ± 10.11 days) had a slightly longer duration than females (28.14 ± 4.95 days) (Table 3).

### 2.4. Local and Systemic Side Effects by Gender

Overall, 85.8% of the participants reported at least one local side effect related to the injection site. Females (88.1%) had a statistically significantly (*χ*^2^ = 7.186; *Sig*. = 0.007) higher prevalence of local side effects than males (78.3%). Similarly, females (1.07 ± 0.62) had a statistically significantly (*U* = 21375; *Sig*. = 0.016) higher intensity of local side effects than males (0.94 ± 0.69). The intensity of the local side effects was defined as the number of side effects per participant, and it ranged between 0 and 3.

The most common local side effect was injection site pain (85.2%), followed by injection site swelling (10.2%) and injection site redness (8.4%). The difference between females (87.3%) and males (78.3%) was only significant (*χ*^2^ = 5.926; *Sig*. = 0.015) in case of the injection site pain.

Overall, 70.5% of the participants reported at least one systemic side effect, with the female participants (75.6%) being more significantly (*χ*^2^ = 22.074; *Sig*. < 0.001) affected than the male participants (53.3%). Additionally, the intensity of systemic side effects was statistically significantly (*U* = 18969.5; *Sig*. < 0.001) higher in females (2.29 ± 2.09) than males (1.62 ± 2.00). The intensity of the systemic side effects was defined as the number of side effects per participant, and it ranged between 0 and 9.

**Figure 2:**
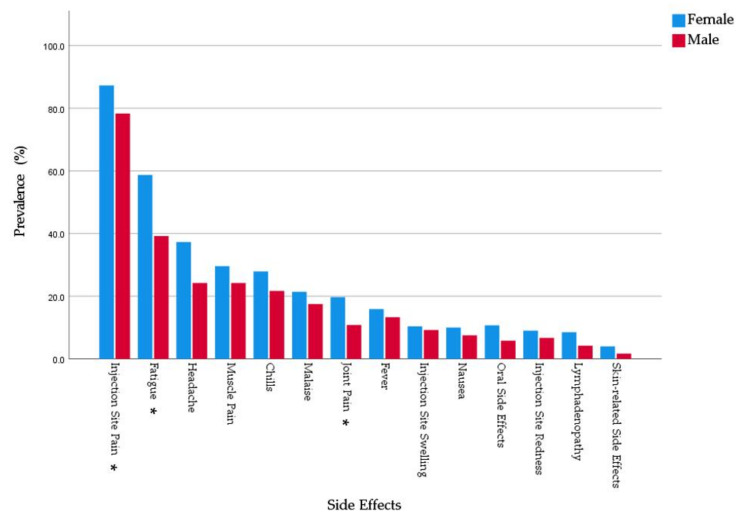
Side effects experienced by Slovak healthcare workers who received BNT162b2 and stratified by gender, February–March 2021 (*n* = 522). * Significance level < 0.05.

The most common systemic side effects were fatigue (54.2%), followed by headache (34.3%), muscle pain (28.4%), chills (26.4%), and malaise (20.5%). Females had higher prevalence of all the solicited systemic side effects in this study. The gender-based differences were statistically significant in case of fatigue (*χ*^2^ = 14.215; *Sig*. < 0.001), headache (*χ*^2^ = 7.089; *Sig*. = 0.008), and joint pain (*χ*^2^ = 4.950; *Sig*. = 0.026) (Figure 2).

In general, 90.4% of the reported side effects resolved within 1–3 days. All males’ side effects were resolved by the 7^th^ day of post-vaccination (100%). In contrast, 3.5% of the females’ side effects lasted for over a week, and 0.5% lasted for more than a month.

The overall prevalence (91.6%) and intensity (3.16 ± 2.33) of post-vaccination side effects were statistically significantly (*χ*^2^ = 6.646 and *U* = 18981; *Sig.* = 0.010 and < 0.001, respectively) higher among females (93.3% and 3.34 ± 2.33) than males (85.8% and 2.57 ± 2.24). The overall intensity was defined as the number of general (local and systemic) side effects reported by a participant, and it ranged between 0 and 12. Only two female participants reported having severe side effects that required seeking medical care (Table 4).

### 2.5. Local and Systemic Side Effects by Age

The young adults (89.9%) had a statistically significantly (*χ*^2^ = 7.591; *Sig*. = 0.022) higher prevalence of local side effects compared to middle-aged (85.2%) and old adults (75%). Similarly, the young adults (1.16 ± 0.69) had a statistically significantly (*H* = 15.091; *Sig.* = 0.001) higher intensity of local side effects than middle-aged (1.01 ± 0.61) and old adults (0.79 ± 0.50). The young adults (89.9% and 14.5%) had statistically significantly (*χ*^2^ = 7.740 and 8.085; *Sig*. = 0.021 and 0.018) higher levels of injection site pain and injection site swelling than middle-aged (84.2% and 8.9%) and old adults (75% and 1.9%).

**Figure 3:**
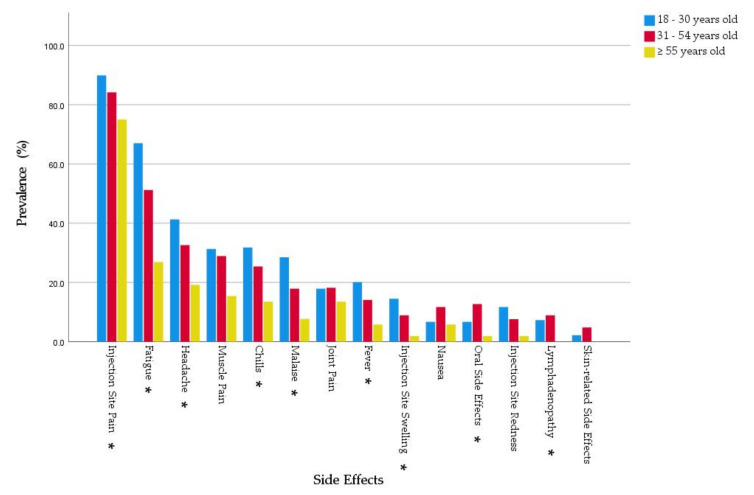
Side effects experienced by Slovak healthcare workers who received BNT162b2 and stratified by age group, February–March 2021 (*n* = 522). * Significance level < 0.05.

Regarding the systemic side effects, young adults (79.3% and 2.52 ± 2.10) had statistically significantly higher levels of prevalence and intensity (*χ*^2^ = 29.385 and *H* = 25.174; *Sig.* = < 0.001 and < 0.001, respectively) compared to middle-aged (70.4% and 2.09 ± 2.08) and old adults (40.4% and 1.08 ± 1.66). All solicited systemic side effects were more common among young adults than middle-aged and old adults except for joint pain, nausea, and lymphadenopathy, where the young and middle-aged groups had a somewhat similar prevalence. The young adults had higher levels of fatigue (67% vs. 26.9%), headache (41.3% vs. 19.2%), fever (20.1% vs. 5.8%), chills (31.8% vs. 13.5%), muscle pain (31.3% vs. 15.4%), joint pain (17.9% vs. 13.5%), malaise (28.5% vs. 7.7%), and lymphadenopathy (7.3% vs. 0%) compared to old adults (Figure 3).

There was no significant difference found in terms of side effects’ duration among the age groups. All of the side effects reported by old adults were resolved within the first week (100%), while 97.6% of the young adults and 96% of the middle-aged adults had their side effects resolved within the first week. Only two of the middle-aged participants reported side effects that persisted for over a week.

The overall prevalence (91.6%) and intensity (3.16 ± 2.33) of post-vaccination side effects were significantly (*2-S Fisher’s Exact Test* and *H* = 30.239; *Sig.* = 0.006 and < 0.001, respectively) higher among young adults (93.9% and 3.68 ± 2.37) and middle-aged adults (92.4% and 3.08 ± 2.29) than old adults (78.8% and 1.87 ± 1.88). Only two middle-aged participants reported having severe side effects that required seeking medical care (Table 5).

### 2.6. Oral Side Effects

Oral side effects were reported by 9.6% of the participants with an overall intensity of 0.14 ± 0.51. The intensity of oral side effects was defined as the number of oral side effects per participant, and it ranged between 0 and 10. The most common oral side effect was burning or bleeding gingiva (3.3%), followed by blisters (2.1%), ulcers (1.9%), and vesicles (1.5%). However, taste disturbance and oral paraesthesia were not solicited in this study; they were reported voluntarily by 0.2% and 0.8% of the participants, thus suggesting that their prevalence was underestimated.

The most common location for ulcers, vesicles, and blisters was labial and buccal mucosa (46.7%), followed by gingiva (33.3%) and tongue (26.7%), while the most common location for the white and red plaque was the tongue (57.1%). Almost one-fifth (20.4%) of the oral side effects emerged within 1–3 days post-vaccination, and another one-fifth (20.4%) emerged within the first week, the second week, and the third week.

The gender-based differences were not statistically significant in terms of oral side effect prevalence, intensity, or onset. Among females, the most common oral side effect was burning or bleeding gingiva (4%), followed by blisters (2%) and ulcers (2%), while among males, the most common oral side effect was blisters (2.5%), followed by ulcers (1.7%) and vesicles (1.7%). Females (10.7% and 0.16 ± 0.54) had statistically insignificantly higher prevalence and intensity (*χ*^2^ = 2.524 and *U* = 22962; *Sig.* = 0.112 and 0.118, respectively) of oral side effects compared to males (5.8% and 0.08 ± 0.36) (Table 6).

The prevalence and intensity of oral side effects were significantly (*2-S Fisher’s Exact Test* and *H* = 8.332; *Sig.* = 0.013 and = 0.016, respectively) higher in middle-aged (12.7% and 0.17 ± 0.51) and young adults (6.7% and 0.12 ± 0.56) than old adults (1.9% and 0.02 ± 0.14). Among middle-aged adults, burning or bleeding gingiva was the most common oral side effect (3.4%), followed by blisters (2.7%), vesicles (2.4%), and halitosis (2.1%). Among young adults, gingiva was the most affected location by ulcers, vesicles, and blisters, while among middle-aged adults, labial and buccal mucosa (54.5%) and tongue (36.4%) were the most common locations (Table 7).

### 2.7. Skin-Related Side Effects

Skin-related side effects were reported by only 3.4% of the participants with an intensity of 0.04 ± 0.22. The intensity of skin-related side effects was defined as the number of skin-related side effects per participant, and it ranged between 0 and 2. Angioedema and skin rash were reported by 2.5% and 1.5% of the participants, respectively. The upper limb was the most common location (61.1%).

While 16 females and 2 males reported skin-related side effects, all gender-based differences were statistically insignificant. The upper limb and torso were the most affected locations among females, while the upper limb and back were the most affected among males (Table 8).

The old adults reported no skin-related side effects. While the young and middle-aged adults had a similar level of skin rash (1.7%), middle-aged adults had more frequent (3.8%) angioedema than young adults (1.1%).

The prevalence and intensity of skin-related side effects were higher among middle-aged adults (4.8% and 0.05 ± 0.26, respectively) than young adults (2.2% and 0.03 ± 0.20, respectively) without statistical significance. The upper limb and torso were the most common location for skin-related side effects reported by young and middle-aged adults (Table 9).

### 2.8. Risk Factors of Side Effects

On running binary logistic regression to evaluate the potential risk factors of mRNA-based vaccine side effects, the demographic variables (gender and age) and anamnestic variables (chronic illnesses, medical treatments, and prior COVID-19 infection) were controlled in order to calculate the adjusted odds ratio (AOR) for each outcome of interest (side effects).

Across gender, females were AOR 2.28 (CI 95%: 1.18–4.44) more likely to experience post-vaccination side effects than their male counterparts. Females had an increased AOR of local side effects 1.97 (1.15–3.39) and systemic side effects with AOR 2.90 (1.86–4.54). The risk of injection site pain, fatigue, headache, and joint pain was statistically significantly (*AOR =* 1.86, 2.35, 1.97, and 1.91; *Sig.* = 0.023, < 0.001, = 0.005, and 0.045, respectively) higher among females than males. In all the other side effects, females had higher AORs while being non-statistically significant.

The young age was another risk factor for post-vaccination side effects; therefore, the old adults were used as a reference group for the logistic regression analysis. The young adults (18–30 years old) were 3.94 (1.51–10.29) times more likely to experience side effects compared to the old adults (≥55 years old). In all the solicited side effects, young adults had increased AORs compared to old adults. The risk of injection site pain, injection site swelling, fatigue, headache, fever, muscle pain, and malaise was statistically significantly (*AOR* = 3.68, 9.05, 6.48, 3, 3.70, 2.45, and 4.71; *Sig.* = 0.002, 0.035, 0.002, <0.001, = 0.006, 0.040, 0.038, and 0.006, respectively) higher among young adults than old adults.

Consequently, the middle-aged adults had an AOR of 2.93 (1.27–6.77) compared to the old adults. The middle-aged adults were AOR 7.56 (1.00–57.08) more likely to experience oral side effects than the old adults. In all the solicited side effects, middle-aged adults had increased AORs compared to old adults. The risk of local and systemic side effects was significantly (*AOR* = 2.14 and 3.54; *Sig.* = 0.047 and <0.001, respectively) higher among middle-aged adults than old adults.

Chronic illnesses did not seem to have a substantial impact on post-vaccination side effects. The AOR of the participants with chronic illnesses was ≈1 in injection site pain, injection site swelling, local side effects, fatigue, chills, joint pain, and systemic side effects. Only muscle pain was 1.88 (1.01–3.53) times more likely to be reported by the participants with chronic illnesses.

The medical treatments were also not very influential in triggering side effects, as they were associated with increased AORs of injection site pain 1.75 (0.77–3.99), injection site swelling 1.26 (0.55–2.90), and injection site redness 2.53 (1.12–5.68). In general, the systemic side effects were less likely to be reported by the participants with regular medications. The medical treatments were associated with decreased AORs of headache 0.75 (0.43–1.31), fever 0.59 (0.27–1.28), chills 0.55 (0.29–1.03), muscle pain 0.61 (0.33–1.11), nausea 0.46 (0.17–1.23), malaise 0.68 (0.35–1.34), oral side effects 0.83 (0.34–2.01), and skin-related side effects 0.75 (0.17–3.34).

Prior COVID-19 infection was associated with slightly increased AORs of local side effects, including injection site pain 1.30 (0.59– 2.89), injection site swelling 1.50 (0.71–3.17), and injection site redness 1.16 (0.49–2.75). Chills were AOR 1.85 (1.08–3.17) more likely to be reported by the participants with prior COVID-19 infection (Table 10).

On analysing the individual chronic illnesses and medical treatments, allergy was not markedly associated with any increase or decrease in prevalence of post-vaccination side effects, except for lymphadenopathy, which was reported by 33.3% of the allergic and 6.9% of the non-allergic participants (*2-S Fisher’s Exact Test*; *Sig.* = 0.009). Most of the solicited systemic side effects in this study were more common among the asthmatic participants, e.g., fatigue (65.4% vs. 53.6%), fever (23.1% vs. 14.9%), malaise (30.8% vs. 20%), muscle pain (50% vs. 27.2%), joint pain (42.3% vs. 16.3%), and lymphadenopathy (15.4% vs. 7.1%).

Local side effects were equally experienced by the participants consuming antihistamine drugs and those who were not. Contrarily, systemic side effects (77.5% vs. 69.9%) tended to be slightly more common among the participants consuming antihistamine drugs, e.g., fatigue (70% vs. 52.9%), fever (20% vs. 14.9%), malaise (25% vs. 20.1%), joint pain (27.5% vs. 16.8%), and lymphadenopathy (22.5% vs. 6.2%). Most of the solicited systemic side effects in this study were more common among the participants consuming analgesics, e.g., fatigue (77.8% vs. 53.8%), headache (55.6% vs. 33.9%), fever (33.3% vs. 15%), chills (44.4% vs. 26.1%), nausea (33.3% vs. 9%), and joint pain (33.3% vs. 17.3%).

## 3. Discussion

The vast majority (91.6%) of the Slovak healthcare workers included in this study reported at least one side effect after receiving an mRNA-based COVID-19 vaccine, BNT162b2; 85.8% experienced local side effects related to the injection site, and 70.5% experienced systemic side effects. All reported side effects were of minor nature and relatively short duration. Injection site pain (85.2%) was the most common local side effect, followed by injection site swelling (10.2%) and injection site redness (8.4%). Fatigue (54.2%) was the most common systemic side effect, followed by headache (34.3%), muscle pain (28.4%), and chills (26.4%). More than half (56.4%) of the solicited side effects lasted only one day, and more than one-third (34%) were resolved within three days.

To date, several independent (non-sponsored) phase IV studies on mRNA-based COVID-19 vaccines have been published from various countries, e.g., Czech Republic, Germany, Greece, Iraq, Italy, Jordan, Malta, Poland, Saudi Arabia, the UK, and the USA [13,19,20,21,22,23,24,25,26,27,28,29,30]. The results of these phase IV studies generally agreed with the results of the phase III trials executed by the manufacturers and published by the regulators, i.e., the Centers for Disease Control and Prevention (CDC) [31,32]. The main domains of agreement between phase III and phase IV studies are the intensity and duration of the side effects, while the domain of disagreement is the prevalence of the side effects and the health status of the recipients, as the phase III trials may tend to have relatively healthier individuals than the average general population [20].

In the Czech Republic, a post-marketing study among healthcare workers who received BNT162b2 revealed that, similar to our findings, injection site pain (89.8%) was the most common local side effect followed by injection site swelling (25.6%) and injection site redness (23%) [19]. The prevalence of injection site swelling (10.2% vs. 25.6%) and injection site redness (8.4% vs. 23%) among our participants was significantly lower than what was reported in the Czech Republic (*χ*^2^ = 39.672 and 39.462; *Sig.* < 0.001 and < 0.001, respectively). Similarly, the Czech healthcare workers reported significantly higher (*χ*^2^ = 8.805, 5.550, 12.360, 5.147, and 3.980; *Sig.* = 0.003, 0.018, < 0.001, = 0.023, and 0.046) prevalence of systemic side effects compared to our Slovak participants, e.g., headache (45.6% vs. 34.3%), muscle pain (37.1% vs. 28.4%), joint pain (27.8% vs. 17.6%), fever (21.7% vs. 15.3%), and chills (33.9% vs. 26.4%), respectively [19]. Moreover, the intensity of the side effects was higher among the Czechs 4.19 ± 2.615 (0–12) than the Slovaks 3.16 ± 2.33 (0–12) [19].

The duration of the side effects reported by Czech and Slovak healthcare workers was quite similar, as 90.3% and 94.8% of their side effects were resolved within five days, respectively. The severe side effects that required medical attention were less frequent among our sample (0.4%) than the Czech sample (1.3%).

Kadali et al. (2021) found that among BNT162b2 recipients, injection site pain (88%), injection site swelling (5.5%), and injection site discolouration (1.25%) were the most common local side effects [22]. They also surveyed a group of healthcare workers in the USA who received mRNA-1273, and they found the same common local side effects but with higher prevalence levels: injection site pain (94.21%), injection site swelling (15.05%), and injection site discolouration (3.47%) [21]. The same trend was found in the CDC report, as BNT162b2 had less prevalent (*χ*^2^ = 507.889, 53.312 and 0.070; *Sig.* < 0.001, < 0.001 and = 0.792) local side effects compared to mRNA-1273: injection site pain (75.5% vs. 85.9%), injection site swelling (6.4% vs. 9%), and injection site redness (5.5% vs. 5.6%), respectively [31,32].

Additionally, the studies of Kadali et al. (2021) confirmed that systemic side effects were more common after mRNA-1273 than BNT162b2, e.g., fatigue (65.7% vs. 58.9%), headache (59.3% vs. 45.5%), muscle pain (54.2% vs. 45.7%), joint pain (24.8% vs. 16.6%), fever (35.7% vs. 22%), chills (52.8% vs. 36.6%), and nausea (26.6% vs. 16%), respectively [21,22]. These results were in agreement with the CDC report, as mRNA-1273 had more prevalent (*χ*^2^ = 14.088, 62.89, 521.734, 630.659 and 103.971; *Sig.* < 0.001, <0.001, <0.001, <0.001 and <0.001) systemic side effects compared to BNT162b2: fatigue (50.6% vs. 48.2%), headache (45.1% vs. 40.1%), muscle pain (39.4% vs. 25.5%), joint pain (29% vs. 15%), and chills (25.3% vs. 19.7%), respectively [31,32]. Although, the cross-vaccine comparison would have been an appealing target for our study, it was not feasible to perform in our sample due to the suboptimal number of mRNA-1273 recipients.

Nevertheless, our sample was not equally distributed across gender, with 77% being females; the Slovak National Health Information Centre (NHIC) revealed in its latest report of 2019 that 78% of Slovak healthcare workers were females, thus confirming the national representativeness of our sample [33]. The gender-based differences of post-vaccination side effect prevalence in our sample indicated the higher susceptibility of females to experience and report side effects. This finding is in line with what was previously reported by phase IV trials of COVID-19 vaccines from Italy, Saudi Arabia, and the UK, where female participants reported side effects more frequently than males [20,28,34] Di Resta et al. (2021) found that Italian female healthcare workers had significantly higher serological values after receiving COVID-19 vaccines, which were correlated with a higher prevalence of post-vaccination side effects, thus suggesting that the more prevalent and severe side effects reported by females might be explained by their potent immune response [34]. The role of sex hormones in immunity can also suggest an explanatory hypothesis for these differences; while testosterone decreases immune functions, the normal levels of oestrogen can stimulate humoral responses to viral infections [35,36]. Moreover, the empirically assumed lower pain threshold of females and the barriers of males to have help-seeking behaviours can further explain the gender-based differences in self-reported COVID-19 vaccine side effects [37,38,39].

The median age of our sample (36 years old) was considerably lower than the median age of the general Slovak population (41 years old). This can be explained by the fact that our target population was healthcare workers who belong entirely to the working-age group (15–64 years old) that represents 68.2% of the Slovak population [40]. Regarding the age structure of our sample, 23.2% of physicians, dentists, and nurses were 18–29 years old, 58.6% were 30–49 years old, and 18.2% were ≥50 years old. According to the Slovak NHIC’s latest report, 9.6% of physicians, dentists, and nurses were 18–29 years old, 48.8% were 30–49 years old, and 41.6% were ≥50 years old, thus indicating that our sample was a bit younger than the actual Slovak healthcare worker population which can be explained by the fact that this study used a digital form to collect the data [41]. According to the United Nations Economic Commission for Europe (UNECE), almost 90% of young and middle-aged adult Slovaks used the Internet on a daily basis compared to only 58% of old adults (≥55 years old) using the Internet daily [42]. Kelfve et al. (2020) found that online surveying might be a feasible option in health studies targeting an old population; however, the paper questionnaire remains an indispensable method to avoid missing subsets of the geriatric population that can bias the final estimates [43].

Age was a significant risk factor of post-vaccination side effect incidence; the younger age groups were associated with an increased adjusted odds of side effects. Menni et al. (2021) found that systemic side effects were significantly more common among the ≤55 years than the >55 years old recipients of BNT162b2 in the UK [20]. Cuschieri et al. (2021) found that the BNT162b2 side effects reported by Maltese healthcare workers were significantly higher among those aged below 45 years old regardless of their sex [27]. Almufty et al. (2021) found that the <50-year-old participants were prone to increased prevalence and intensity of post-vaccination side effects in Iraq [24]. In the Czech Republic, the local and systemic side effects of BNT162b2 were significantly more common among healthcare workers aged 43 years old or below [19].

The age-dependent differences were not an unpredicted finding recorded by the post-marketing studies; they had been already reported by the manufacturers in the phase III trials [31,32]. In the CDC report of BNT162b2, people aged above 55 years had less prevalent side effects than those who were aged 55 years or below, e.g., injection site pain (68.7% vs. 80.6%), fatigue (42% vs. 53.1%), headache (31.8% vs. 46.6%), muscle pain (21% vs. 28.9%), joint pain (13.5% vs. 16.2%), fever (6% vs. 9.5%), chills (14.2% vs. 24.1%), and vomiting (0.6% vs. 1.5%), respectively [31]. In the CDC report of mRNA-1273, people aged above 64 years had less prevalent side effects than those who were aged 64 years or below, e.g., injection site pain (78.5% vs. 88.4%), injection site swelling (7.5% vs. 9.5%), injection site redness (4.8% vs. 5.9%), fatigue (45.5% vs. 52.3%), headache (35.2% vs. 48.4%), muscle pain (33% vs. 41.6%), joint pain (25.4% vs. 30.2%), fever (5.1% vs. 8.8%), chills (17.7% vs. 27.8%), and nausea (8.4% vs. 15.1%), respectively [32].

Sprent et al. (2021) suggested that the higher prevalence and intensity of COVID-19 vaccine side effects reported by young adults and females can be explained by the concomitant production of type-I interferon (INF-I) with the effective immune response [44,45]. The levels of INF-1 in young adults were found to be higher following COVID-19 vaccination than after COVID-19 infection; thus, also explaining why young adults tend not to exhibit severe symptoms following infection, while they have stronger side effects following vaccination [44].

The impact of chronic illnesses on side effect incidence has not yet been thoroughly investigated; therefore, one of the strongest points of this study is the exhaustive inquiry about participants’ medical anamneses. Our sample participants with chronic illnesses had slightly lower adjusted odds of local side effects and slightly higher adjusted odds of systemic side effects. On the one hand, the adverse events after vaccination may be intensified due to any underlying medical conditions, or they may reflect a coincidental novel condition; on the other hand, a possible reduced immune capacity due to some pre-existing illnesses may lead to a less vigorous immune response and less prevalent side effects [21]. These hypotheses can be carried on while attempting to understand the role of medical treatments in post-vaccination side effect incidence and intensity.

Nevertheless, little is known about the drug–drug interaction of COVID-19 vaccines with other medications. Kow et al. (2021) suggested that there might be interactions between COVID-19 vaccines (mRNA-based and viral vector-based ones) with antiepileptic drugs due to the interferon-gamma production elicited by the vaccines, thus warranting active vigilance by clinicians and epileptic patients for post-vaccination events [46]. In our sample, there were only two participants who reported using antiepileptic drugs; therefore, subgroup analysis was not deemed possible to verify the assumptions of Kow et al. (2021) in terms of side effect incidence and intensity.

In the Czech Republic, antihistamines were associated with increased adjusted odds of several side effects, e.g., injection site redness, headache, nausea, fever, chills, and lymphadenopathy [19]. Similarly, our participants consuming antihistamines had slightly more frequent systemic side effects, e.g., fatigue, fever, malaise, joint pain, and lymphadenopathy. A recent multi-centre retrospective study evaluated individuals who experienced immediate allergic reactions after the first dose of mRNA-based COVID-19 vaccines. All the patients who received the second dose tolerated it, and 30% of them used antihistamine premedication to decrease the odds of experiencing severe allergic reactions after the second dose [47]. The chronic use of antihistamines, as in our sample, can be to manage certain allergic conditions that would not necessarily increase the odds of allergic reactions after vaccination [48].

The oral cavity represents a vital locus for exhibiting extrapulmonary symptoms of SARS-CoV-2 [49,50,51]. The oral manifestations of COVID-19 patients varied significantly, and they included dysgeusia, xerostomia, aphthous stomatitis, herpetic ulcers, oral mucositis, salivary gland involvement, and fungal co-infections such as oral candidiasis and mucormycosis [49,52,53,54,55,56,57,58,59,60]. However, the pathophysiology of these symptoms is not fully understood, and a number of hypotheses have been proposed to explain them, including inflammatory response; the direct infiltration of SARS-CoV-2 to the lining epithelium of the oral cavity, which was found to be rich with angiotensin-converting enzyme 2 (ACE2) receptors; and secondary infection [49,50]. Given the proposition that these oral manifestations are immune-dependent, the possibility of their emergence after receiving COVID-19 vaccines cannot be omitted. Therefore, we included the oral side effects within our inquiry for rare side effects.

Menni et al. (2021) found that red welts on lips were reported by 0.2% of the recently vaccinated British individuals following both doses of mRNA-based vaccines [20]. In the first case series of severe reactions following the first dose of BNT162b2 in the USA, swollen tongue and swollen lip were reported in 9.5% and 19% of the reported cases of anaphylactic shock, respectively [61]. The studies of Kadali et al. (2021) found that swollen lips and tongue were reported by 0.12% and 0.23% of BNT162b2 and mRNA-1273 recipients, respectively [21,22]. The allergy to mRNA-based vaccine ingredients, especially Poly(ethylene glycol) (PEG), can be associated with the incidence of severe ulcerative lesions of the oral mucosa and inflamed lips, as reported in the recent case of Manfredi et al. (2021) from Italy [62].

### 3.1. Strengths

To the best of the authors’ knowledge, this study is the first to evaluate COVID-19 vaccine side effects among the Slovak population in the post-marketing stage. It is also among the earliest studies to compare side effect prevalence, intensity, and duration among different age groups with a highlight of young adults (18–30 years old) who represent a critical target for mass vaccination strategies globally to achieve herd immunity. Inquiring about uncommon side effects such as oral and skin-related ones is another strong point of our study, as these side effects are usually overlooked, while they might be worrisome for the vaccinated individuals despite their mild nature.

The potential adjusted risk factors of mRNA-based COVID-19 vaccines were analysed for a better understanding of the role of gender, age, and prior COVID-19 infection in side effect incidence and intensity. We surveyed an extensive list of chronic illnesses and medical treatments in order to evaluate their association with post-vaccination side effects. The recruited sample was intended to be homogenous by having similar levels of health literacy and by being healthcare workers, by belonging to the working-age population, and by receiving both doses of the vaccine as an inclusion criterion.

### 3.2. Limitations

The first limitation of this study is the use of self-reported data, similar to all other phase IV trials; therefore, healthcare workers were chosen to be the target population as they are deemed to have the highest possible levels of health literacy and scientific interest. The second limitation is the predominance of the BNT162b2 COVID-19 vaccine at the expenses of the mRNA-1273 COVID-19 vaccine in our sample, which could be explained by the actual distribution levels of both vaccines among the target population.

The third limitation is related to the structure of the instrument used, as we did not inquire about the onset of each side effect and whether side effects occurred after the first dose, the second dose, or both doses. The duration of side effects was not evaluated individually to simplify the questionnaire and warrant a sufficient number of responses. The last limitation is the lack of information about the response rate of the study, as the digital platform used, KoboToolbox, does not enable the researchers to learn the number of the form visitors who represent the denominator of the response rate equation.

### 3.3. Implications

The findings of this study warrant further investigation for the gender-based and age-based differences in COVID-19 vaccine side effects. Future research on COVID-19 vaccine safety should also precisely evaluate the onset and duration of mRNA-based COVID-19 vaccine side effects in relation to their potential demographic and medical risk factors.

The safety profile of viral vector-based COVID-19 vaccines among the Slovak population should be included in future studies, as a cross-vaccine comparison might be needed by policymakers and individuals to make evidence-informed decisions. The execution and reporting of COVID-19 vaccine safety studies should follow a standardised methodology; therefore, multi-centre studies with similar methods and concise reporting guidelines are deemed required at this stage [17].

## 4. Materials and Methods

### 4.1. Design

An independent (non-sponsored) phase IV study was carried out to evaluate the post-vaccination side effects of mRNA-based COVID-19 vaccines experienced by healthcare workers in Slovakia. The study utilised a validated self-administered questionnaire (SAQ) coded and disseminated online through KoBoToolbox version 2.021.03 (Harvard Humanitarian Initiative. Cambridge, MA, USA, 2021) [63]. The protocol had been registered a priori at the US National Library of Medicine (NLM) with the identifier NCT04706156, and the study was conducted and reported according to the STROBE guidelines for cross-sectional studies [64,65].

### 4.2. Participants

The target population was healthcare workers who received COVID-19 vaccines during “Phase 1” of the mass vaccination strategy timeline determined by the Slovak MoH decree [6]. The first inclusion criterion was to have received an mRNA-based COVID-19 vaccine, either BNT162b2 or mRNA-1273, as these vaccines were the only authorised vaccines by the European Medicines Agency (EMA) when Phase I was initiated in December 2020. The second inclusion criterion was to have received both doses of the vaccine. The participants did not receive financial rewards, and their participation was not incentivised by any other means of compensation to control selection and information biases.

Non-random sampling through a snowballing technique was utilised in order to recruit the target population. The recruitment process took place during February and March 2021, and the participants were invited through distribution lists of hospitals in Banská Bystrica and Bratislava. The social media groups of medical and healthcare workers were also used as auxiliary recruitment channels, in addition to word-of-mouth promotion.

**Figure 4:**
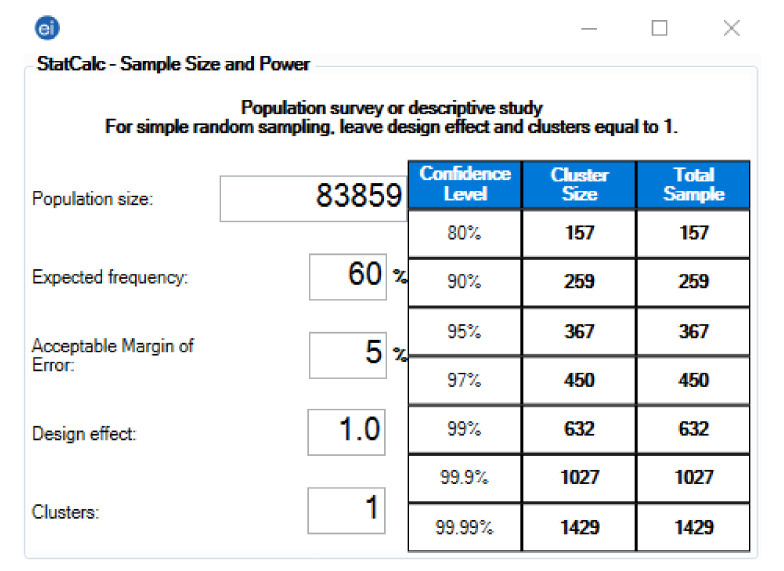
Sample size of the Slovak healthcare workers: Epi Info^TM^ version 7.2.4. Population Size: According to the latest report of the National Health Information Centre (NHIC), there were 83,859 healthcare workers in Slovakia by 2019 [33]. Expected Frequency: The overall prevalence of side effects following COVID-19 vaccines ranged between 62% and 93% [19,67,68,69]. Acceptable Error Margin: 5% was assumed permissible because the expected frequency ranged between 10% and 90% [70]. Design Effect: 1–per the recommendation of the CDC for simple sampling [71]. Clusters: 1–per the recommendation of the CDC for simple sampling [71].

The required sample size was calculated using Epi Info^TM^ version 7.2.4 (CDC, Atlanta, GA, USA, 2020) [66]. The following assumption was used in the population survey (descriptive study) module: expected outcome frequency of 60%, error margin of 5%, and confidence level of (*CI*) 95%. Given that the target population size was 83,859, the minimum sample should be 404 participants, including a 10% predicted no-response rate (Figure 4).

### 4.3. Instrument

The instrument of this study was adapted in accordance with the results of phase III trials, and it was validated before being translated into Slovak language. The psychometric properties and the whole validation process are described in detail elsewhere [19]. First, a panel of experts checked the content validity of the proposed SAQ, then a group of volunteer healthcare workers filled it twice to evaluate the test re-test reliability, yielding substantial reliability with a Cohen’s kappa coefficient of 0.89 ± 0.13 (0.54–1) [19].

The translation process involved two basic steps: (a) dual forward translation from English to Slovak by two independent translators, and (b) a panel of experts’ review of the two draft versions and their comparison to resolve any discrepancies and produce a coherent final version [72].

The SAQ consisted of twenty mandatory multiple-choice items and eight conditional multiple-choice items, and it was divided into four categories: (a) demographic information including gender, age, region, profession, and length of work experience; (b) medical anamneses including chronic illnesses and medical treatments; (c) COVID-19-related anamneses including prior infection, contact with confirmedly infected cases, vaccine type, number of vaccine doses, and their dates; and (d) the prevalence of local, systemic, oral, and skin-related side effects, their duration, onset, and location.

### 4.4. Ethics

The Ethics Committee of the Faculty of Medicine, Masaryk University, reviewed the study protocol thoroughly and approved it on 20 January 2021, with Ref No. 2/2021. Ethical approval was also obtained from the Ethics Committee of F. D. Roosevelt University Hospital on 23 February 2021, with Ref No. 8/2021. The study was carried out according to the Declaration of Helsinki for research on human subjects, and all the participants had to give their informed consent digitally before filling in the questionnaire [73].

Participation was entirely voluntary, and the participants had the right to withdraw at any time before submitting their answers without the need to justify their decision. There was no personal data collected that might enable the retrospective identification of the participants. The study data were stored and processed by Masaryk University in full compliance with the European Union (EU) General Data Protection Directive (GDPR) [74].

### 4.5. Analysis

All the descriptive and inferential tests were carried out using the Statistical Package for the Social Sciences (SPSS) version 27 (SPSS Inc. Chicago, IL, USA, 2020) [75]. The normal distribution of the dependent variables was evaluated through Shapiro–Wilk test with a significance level (*Sig.*) of 0.05.

Initially, descriptive statistics were executed using frequencies (*n*), percentages (%), means, and standard deviations (*μ ± SD*) to present the demographic variables, medical anamneses, COVID-19 related anamneses, and side effect prevalence, duration, onset, and location. Afterwards, inferential statistics were executed using the chi-squared test (*χ*^2^), Fisher’s exact test, Mann–Whitney test (*U*), and Kruskal–Wallis test (*H*) to evaluate the association between the demographic, medical, and COVID-19-related variables (independent variables) and post-vaccination side effects (dependent variables).

Finally, binary logistic regression was used to evaluate the potential demographic and medical risk factors (predictors) of post-vaccination side effects and to estimate the adjusted odds ratio (AOR). All inferential tests were considered significant at the level of < 0.05.

## 5. Conclusions

Most of the Slovak healthcare workers (91.6%) who received mRNA-based COVID-19 vaccines, BNT162b2, reported at least one side effect. In agreement with the previous phase III and IV studies, injection site pain was the most common local side effect, and fatigue, headache, muscle pain, and chills were the most common systemic side effects. The reported side effects were of a mild nature (99.6%) that did not require medical attention and a short duration, as most of them (90.4%) were resolved within three days.

Females and young adults (18–30 years old) were more likely to report post-vaccination side effects; such a finding is also consistent with what was recently reported in various parts of the world. The role of chronic illnesses and medical treatments in post-vaccination side effect incidence and intensity requires further robust investigation among large population groups. Future research on COVID-19 vaccine safety should benefit from a standardised methodology for execution and reporting to facilitate cross-vaccine comparison.

## Figures and Tables

**Table 1:** Demographic characteristics of Slovak healthcare workers who received BNT162b2, February–March 2021 (*n* = 522).

Variable	Outcome	Frequency (*n*)	Percentage (%)
Gender	Female	402	77%
	Male	120	23%
Age	18–30 years old	179	34.3%
	31–54 years old	291	55.7%
	≥55 years old	52	10%
Profession	Physician	245	46.9%
	Dentist	46	12.3%
	Nurse	97	18.6%
	Midwife	6	1.1%
	Nursing Assistant	59	11.3%
	Paramedic	7	1.3%
	Lab Worker	10	1.9%
	Pharmacist	4	0.8%
	Psychologist	4	0.8%
	Dietitian	2	0.4%
	Dental Hygienist	2	0.4%
	Administrative Staff	15	2.9%
	Other	7	1.3%
Experience	1–5 years	190	36.4%
	6–10 years	83	15.9%
	11–20 years	103	19.7%
	>20 years	146	28%
Region	Banská Bystrica	217	41.6%
	Bratislava	102	19.5%
	Košice	38	7.3%
	Nitra	31	5.9%
	Prešov	34	6.5%
	Trenčín	18	3.4%
	Trnava	33	6.3%
	Žilina	49	9.4%

**Table 2:** Medical anamnesis of Slovak healthcare workers who received BNT162b2, February–March 2021 (*n* = 522).

Variable	Outcome	Female (*n* = 402)	Male (*n* = 120)	Total (*n* = 522)	*Sig.*
Chronic Illness	Allergy	8 (2%)	4 (3.3%)	12 (2.3%)	0.485
	Asthma	21 (5.2%)	5 (4.2%)	26 (5%)	0.640
	Blood Disease	2 (0.5%)	0 (0%)	2 (0.4%)	1.000
	Bone Disease	2 (0.5%)	0 (0%)	2 (0.4%)	1.000
	Bowel Disease	13 (3.2%)	4 (3.3%)	17 (3.3%)	1.000
	Cancer	3 (0.7%)	1 (0.8%)	4 (0.8%)	1.000
	Cardiac Disease	5 (1.2%)	3 (2.5%)	8 (1.5%)	0.393
	Chronic Hypertension	26 (6.5%)	10 (8.3%)	36 (6.9%)	0.479
	COPD	1 (0.2%)	0 (0%)	1 (0.2%)	1.000
	Diabetes Mellitus	6 (1.5%)	1 (0.8%)	7 (1.3%)	1.000
	Kidney Disease	2 (0.5%)	0 (0%)	2 (0.4%)	1.000
	Neurological Disease	4 (1%)	1 (0.8%)	5 (1%)	1.000
	Psychological Distress	2 (0.5%)	0 (0%)	2 (0.4%)	1.000
	Rheumatoid Arthritis	4 (1%)	0 (0%)	4 (0.8%)	0.578
	Thyroid Disease	33 (8.2%)	1 (0.8%)	34 (6.5%)	0.004
	Other	5 (1.2%)	2 (1.7%)	7 (1.3%)	0.663
	Total	108 (26.9%)	27 (22.5%)	135 (25.9%)	0.338
Medical Treatment	Analgesics	9 (2.2%)	0 (0%)	9 (1.7%)	0.127
	Anticoagulants	1 (0.2%)	1 (0.8%)	2 (0.4%)	0.407
	Antidepressants	8 (2%)	1 (0.8%)	9 (1.7%)	0.692
	Antidiabetics	5 (1.2%)	1 (0.8%)	6 (1.1%)	1.000
	Antiepileptics	2 (0.5%)	0 (0%)	2 (0.4%)	1.000
	Anti-GERD	2 (0.5%)	4 (3.3%)	6 (1.1%)	0.027
	Antihistamines	33 (8.2%)	7 (5.8%)	40 (7.7%)	0.391
	Antihypertensives	37 (9.2%)	13 (10.8%)	50 (9.6%)	0.595
	Cholesterol-lowering	1 (0.2%)	0 (0%)	1 (0.2%)	1.000
	Contraceptives	5 (1.2%)	0 (0%)	5 (1%)	0.594
	Immunosuppressives	14 (3.5%)	5 (4.2%)	19 (3.6%)	0.781
	NSAID	14 (3.5%)	1 (0.8%)	15 (2.9%)	0.210
	Thyroid Hormones	39 (9.7%)	1 (0.8%)	40 (7.7%)	0.001
	Other	7 (1.7%)	0 (0%)	7 (1.3%)	0.361
	Total	138 (34.3%)	28 (23.3%)	166 (31.8%)	0.023

Chi-squared test (*χ*^2^) and Fisher’s exact test were used with a significance level (*Sig.*) of < 0.05.

**Table 3:** COVID-19-related anamnesis of Slovak healthcare workers who received BNT162b2, February–March 2021 (*n* = 522).

Variable	Outcome	Female (*n* = 402)	Male (*n* = 120)	Total (*n* = 522)	*Sig.*
Prior Infection	Yes	53 (13.2%)	16 (13.3%)	69 (13.2%)	0.966
	No	349 (86.8%)	104 (86.7%)	453 (86.8%)	
Prior Contact	Yes	232 (57.7%)	86 (71.7%)	318 (60.9%)	0.006
	No	170 (42.3%)	34 (28.3%)	204 (39.1%)	
Duration	2nd Dose–1st Dose	28.14 ± 4.95	29.55 ± 10.11	28.46 ± 6.52	0.027

Chi-squared test (*χ*^2^) and Mann–Whitney test (*U*) were used with a significance level (*Sig.*) of < 0.05.

**Table 4:** Local and systemic side effects of Slovak healthcare workers who received BNT162b2 and stratified by gender, February–March 2021 (*n* = 522).

Variable	Outcome	Female (*n* = 402)	Male (*n* = 120)	Total (*n* = 522)	*Sig.*
Local Side Effects	Injection Site Pain	351 (87.3%)	94 (78.3%)	445 (85.2%)	0.015
	Injection Site Swelling	42 (10.4%)	11 (9.2%)	53 (10.2%)	0.683
	Injection Site Redness	36 (9%)	8 (6.7%)	44 (8.4%)	0.428
	Intensity (0–3)	1.07 ± 0.62	0.94 ± 0.69	1.04 ± 0.64	0.016
	Total (*n*)	354 (88.1%)	94 (78.3%)	448 (85.8%)	0.007
Systemic Side Effects	Fatigue	236 (58.7%)	47 (39.2%)	283 (54.2%)	<0.001
	Headache	150 (37.3%)	29 (24.2%)	179 (34.3%)	0.008
	Fever	64 (15.9%)	16 (13.3%)	80 (15.3%)	0.490
	Chills	112 (27.9%)	26 (21.7%)	138 (26.4%)	0.177
	Muscle Pain	119 (29.6%)	29 (24.2%)	148 (28.4%)	0.246
	Joint Pain	79 (19.7%)	13 (10.8%)	92 (17.6%)	0.026
	Nausea	40 (10%)	9 (7.5%)	49 (9.4%)	0.419
	Malaise	86 (21.4%)	21 (17.5%)	107 (20.5%)	0.354
	Lymphadenopathy	34 (8.5%)	5 (4.2%)	39 (7.5%)	0.117
	Intensity (0–9)	2.29 ± 2.09	1.62 ± 2.00	2.14 ± 2.08	<0.001
	Total	304 (75.6%)	64 (53.3%)	368 (70.5%)	<0.001
Side Effects Duration	One Day	206 (54.9%)	63 (61.8%)	269 (56.4%)	0.809
	Three Days	128 (34.1%)	34 (33.3%)	162 (34%)	0.466
	Five Days	17 (4.5%)	4 (3.9%)	21 (4.4%)	0.796
	One Week	9 (2.4%)	1 (1%)	10 (2.1%)	0.467
	>One Week	13 (3.5%)	0 (0%)	13 (2.7%)	0.046
	>One Month	2 (0.5%)	0 (0%)	2 (0.4%)	1.000
General Side Effects	Intensity (0–12)	3.34 ± 2.33	2.57 ± 2.24	3.16 ± 2.33	<0.001
	Total	375 (93.3%)	103 (85.8%)	478 (91.6%)	0.010
Severe Side Effects	Total	2 (0.5%)	0 (0%)	2 (0.4%)	1.000

Chi-squared test (*χ*^2^), Fisher’s exact test, and Mann–Whitney test (*U*) were used with a significance level (*Sig.*) of < 0.05.

**Table 5:** Local and systemic side effects of Slovak healthcare workers who received BNT162b2 and stratified by age group, February–March 2021 (*n* = 522).

Variable	Outcome	18–30 Years Old (*n* = 179)	31–54 Years Old (*n* = 291)	≥55 Years Old (*n* = 52)	Total (*n* = 522)	*Sig.*
Local SE	Injection Site Pain	161 (89.9%)	245 (84.2%)	39 (75%)	445 (85.2%)	0.021
	Injection Site Swelling	26 (14.5%)	26 (8.9%)	1 (1.9%)	53 (10.2%)	0.018
	Injection Site Redness	21 (11.7%)	22 (7.6%)	1 (1.9%)	44 (8.4%)	0.058
	Intensity (0–3)	1.16 ± 0.69	1.01 ± 0.61	0.79 ± 0.50	1.04 ± 0.64	0.001
	Total (*n*)	161 (89.9%)	248 (85.2%)	39 (75%)	448 (85.8%)	0.022
Systemic SE	Fatigue	120 (67%)	149 (51.2%)	14 (26.9%)	283 (54.2%)	< 0.001
	Headache	74 (41.3%)	95 (32.6%)	10 (19.2%)	179 (34.3%)	0.009
	Fever	36 (20.1%)	41 (14.1%)	3 (5.8%)	80 (15.3%)	0.028
	Chills	57 (31.8%)	74 (25.4%)	7 (13.5%)	138 (26.4%)	0.025
	Muscle Pain	56 (31.3%)	84 (28.9%)	8 (15.4%)	148 (28.4%)	0.078
	Joint Pain	32 (17.9%)	53 (18.2%)	7 (13.5%)	92 (17.6%)	0.705
	Nausea	12 (6.7%)	34 (11.7%)	3 (5.8%)	49 (9.4%)	0.156
	Malaise	51 (28.5%)	52 (17.9%)	4 (7.7%)	107 (20.5%)	0.001
	Lymphadenopathy	13 (7.3%)	26 (8.9%)	0 (0%)	39 (7.5%)	0.048
	Intensity (0–9)	2.52 ± 2.10	2.09 ± 2.08	1.08 ± 1.66	2.14 ± 2.08	<0.001
	Total	142 (79.3%)	205 (70.4%)	21 (40.4%)	368 (70.5%)	<0.001
SE Duration	One Day	94 (56.3%)	151 (56.1%)	24 (58.5%)	269 (56.4%)	0.710
	Three Days	62 (37.1%)	86 (32%)	14 (34.1%)	162 (34%)	0.408
	Five Days	3 (1.8%)	16 (5.9%)	2 (4.9%)	21 (4.4%)	0.110
	One Week	4 (2.4%)	5 (1.9%)	1 (2.4%)	10 (2.1%)	0.897
	>One Week	4 (2.4%)	9 (3.3%)	0 (0%)	13 (2.7%)	0.544
	>One Month	0 (0%)	2 (0.7%)	0 (0%)	2 (0.4%)	0.617
General SE	Intensity (0–12)	3.68 ± 2.37	3.08 ± 2.29	1.87 ± 1.88	3.16 ± 2.33	<0.001
	Total	168 (93.9%)	269 (92.4%)	41 (78.8%)	478 (91.6%)	0.006
Severe SE	Total	0 (0%)	2 (0.7%)	0 (0%)	2 (0.4%)	0.617

Chi-squared test (*χ*^2^), Fisher’s exact test, and Kruskal–Wallis test (*H*) were used with a significance level (*Sig.*) of < 0.05.

**Table 6:** Oral side effects of Slovak healthcare workers who received BNT162b2 and stratified by gender, February–March 2021 (*n* = 522).

Variable	Outcome	Female (*n* = 402)	Male (*n* = 120)	Total (*n* = 522)	*Sig.*
Oral Side Effect Prevalence	Ulcers	8 (2%)	2 (1.7%)	10 (1.9%)	1.000
	Vesicles	6 (1.5%)	2 (1.7%)	8 (1.5%)	1.000
	Blisters	8 (2%)	3 (2.5%)	11 (2.1%)	1.000
	White/Red Plaque	6 (1.5%)	1 (0.8%)	7 (1.3%)	1.000
	Angular Cheilitis	6 (1.5%)	0 (0%)	6 (1.1%)	0.345
	Halitosis	6 (1.5%)	1 (0.8%)	7 (1.3%)	1.000
	Xerostomia	2 (0.5%)	0 (0%)	2 (0.4%)	1.000
	Burning/Bleeding Gingiva	16 (4%)	1 (0.8%)	17 (3.3%)	0.139
	Taste Disturbance	1 (0.2%)	0 (0%)	1 (0.2%)	1.000
	Oral Paraesthesia	4 (1%)	0 (0%)	4 (0.8%)	0.578
	Intensity (0–10)	0.16 ± 0.54	0.08 ± 0.36	0.14 ± 0.51	0.118
	Total (*n*)	43 (10.7%)	7 (5.8%)	50 (9.6%)	0.112
Oral Side Effect Onset	1–3 days	9 (21.4%)	1 (14.3%)	10 (20.4%)	0.467
	1st Week	8 (19%)	2 (28.6%)	10 (20.4%)	1.000
	2nd Week	9 (21.4%)	1 (14.3%)	10 (20.4%)	0.467
	3rd Week	11 (26.2%)	2 (28.6%)	13 (26.5%)	0.742
	4th Week	5 (11.9%)	1 (14.3%)	6 (12.2%)	1.000
Ulcers, Vesicles, and Blisters Location (*n* = 15)	Tongue	4 (36.4%)	0 (0%)	4 (26.7%)	0.516
	Palate	2 (18.1%)	1 (25%)	3 (20%)	1.000
	Labial/Buccal Mucosa	6 (54.5%)	1 (25%)	7 (46.7%)	0.569
	Gingiva	3 (27.3%)	2 (50%)	5 (33.3%)	0.560
	Lips	1 (9.1%)	1 (25%)	2 (13.3%)	0.476
White or Red Plaque Location (*n* = 7)	Tongue	4 (66.7%)	0 (0%)	4 (57.1%)	0.429
	Soft Palate	1 (16.7%)	1 (100%)	2 (28.6%)	0.286
	Labial/Buccal Mucosa	2 (33.3%)	0 (0%)	2 (28.6%)	1.000

Chi-squared test (*χ*^2^), Fisher’s exact test, and Mann–Whitney test (*U*) were used with a significance level (*Sig.*) of < 0.05.

**Table 7:** Oral side effects of Slovak healthcare workers who received BNT162b2 and stratified by age group, February–March 2021 (*n* = 522).

Variable	Outcome	18–30 Years Old (*n* = 179)	31–54 Years Old (*n* = 291)	≥55 Years Old (*n* = 52)	Total (*n* = 522)	*Sig.*
Oral Side Effect Prevalence	Ulcers	3 (1.7%)	7 (2.4%)	0 (0%)	10 (1.9%)	0.714
	Vesicles	3 (1.7%)	5 (1.7%)	0 (0%)	8 (1.5%)	1.000
	Blisters	3 (1.7%)	8 (2.7%)	0 (0%)	11 (2.1%)	0.599
	White/Red Plaque	2 (1.1%)	5 (1.7%)	0 (0%)	7 (1.3%)	0.863
	Angular Cheilitis	3 (1.7%)	3 (1%)	0 (0%)	6 (1.1%)	0.829
	Halitosis	1 (0.6%)	6 (2.1%)	0 (0%)	7 (1.3%)	0.350
	Xerostomia	0 (0%)	2 (0.7%)	0 (0%)	2 (0.4%)	0.617
	Burning/Bleeding Gingiva	6 (3.4%)	10 (3.4%)	1 (1.9%)	17 (3.3%)	1.000
	Taste Disturbance	0 (0%)	1 (0.3%)	0 (0%)	1 (0.2%)	1.000
	Oral Paraesthesia	1 (0.6%)	3 (1%)	0 (0%)	4 (0.8%)	1.000
	Intensity (0–10)	0.12 ± 0.56	0.17 ± 0.51	0.02 ± 0.14	0.14 ± 0.51	0.016
	Total (*n*)	12 (6.7%)	37 (12.7%)	1 (1.9%)	50 (9.6%)	0.013
Oral Side Effect Onset	1–3 days	4 (33.3%)	6 (16.7%)	0 (0%)	10 (20.4%)	0.802
	1st Week	2 (16.7%)	8 (22.2%)	0 (0%)	10 (20.4%)	0.385
	2nd Week	1 (8.3%)	9 (25%)	0 (0%)	10 (20.4%)	0.125
	3rd Week	3 (25%)	9 (25%)	1 (100%)	13 (26.5%)	0.772
	4th Week	2 (16.7%)	4 (11.4%)	0 (0%)	6 (12.2%)	1.000
Ulcers, Vesicles, and Blisters Location (*n* = 15)	Tongue	0 (0%)	4 (36.4%)	0 (0%)	4 (26.7%)	0.516
	Palate	1 (25%)	2 (18.2%)	0 (0%)	3 (20%)	1.000
	Labial/Buccal Mucosa	1 (25%)	6 (54.5%)	0 (0%)	7 (46.7%)	0.569
	Gingiva	3 (75%)	2 (18.2%)	0 (0%)	5 (33.3%)	0.077
	Lips	0 (0%)	2 (18.2%)	0 (0%)	2 (13.3%)	1.000
White or Red Plaque Location (*n* = 7)	Tongue	0 (0%)	4 (80%)	0 (0%)	4 (57.1%)	0.143
	Soft Palate	1 (50%)	1 (20%)	0 (0%)	2 (28.6%)	1.000
	Labial/Buccal Mucosa	1 (50%)	1 (20%)	0 (0%)	2 (28.6%)	1.000

Chi-squared test (*χ*^2^), Fisher’s exact test, and Kruskal–Wallis test (*H*) were used with a significance level (*Sig.*) of < 0.05.

**Table 8:** Skin-related side effects of Slovak healthcare workers who received BNT162b2 and stratified by gender, February–March 2021 (*n* = 522).

Variable	Outcome	Female (*n* = 402)	Male (*n* = 120)	Total (*n* = 522)	*Sig.*
Skin-related Side Effect Prevalence	Rash	7 (1.7%)	1 (0.8%)	8 (1.5%)	0.689
	Angioedema	11 (2.7%)	2 (1.7%)	13 (2.5%)	0.742
	Intensity (0–3)	0.04 ± 0.23	0.03 ± 0.20	0.04 ± 0.22	0.228
	Total (*n*)	16 (4%)	2 (1.7%)	18 (3.4%)	0.390
Skin-related Side Effect Location (*n* = 18)	Face	5 (31.3%)	0 (0%)	5 (27.8%)	1.000
	Upper Limb	9 (56.3%)	2 (100%)	11 (61.1%)	0.497
	Lower Limb	2 (12.5%)	1 (50%)	3 (16.7%)	0.314
	Torso	6 (37.5%)	1 (50%)	7 (38.9%)	1.000
	Back	2 (12.5%)	2 (100%)	4 (22.2%)	0.039

Fisher’s exact test and Mann–Whitney test (*U*) were used with a significance level (*Sig.*) of < 0.05.

**Table 9:** Skin-related side effects of Slovak healthcare workers who received BNT162b2 and stratified by age group, February–March 2021 (*n* = 522).

Variable	Outcome	18–30 Years Old (*n* = 179)	31–54 Years Old (*n* = 291)	≥55 Years Old (*n* = 52)	Total (*n* = 522)	*Sig.*
Skin-related Side Effect Prevalence	Rash	3 (1.7%)	5 (1.7%)	0 (0%)	8 (1.5%)	1.000
	Angioedema	2 (1.1%)	11 (3.8%)	0 (0%)	13 (2.5%)	0.153
	Intensity (0–3)	0.03 ± 0.20	0.05 ± 0.26	-	0.04 ± 0.22	0.120
	Total (*n*)	4 (2.2%)	14 (4.8%)	0 (0%)	18 (3.4%)	0.132
Skin-related Side Effect Location (*n* = 18)	Face	1 (25%)	4 (28.6%)	0 (0%)	5 (27.8%)	1.000
	Upper Limb	2 (50%)	9 (64.3%)	0 (0%)	11 (61.1%)	1.000
	Lower Limb	0 (0%)	3 (21.4%)	0 (0%)	3 (16.7%)	1.000
	Torso	2 (50%)	5 (35.7%)	0 (0%)	7 (38.9%)	1.000
	Back	0 (0%)	4 (28.6%)	0 (0%)	4 (22.2%)	0.524

Fisher’s exact test and Kruskal–Wallis test (*H*) were used with a significance level (*Sig.*) of < 0.05.

**Table 10:** Adjusted risk factors of side effects experienced by Slovak healthcare workers who received BNT162b2, February–March 2021 (*n* = 522).

		Gender Female (vs. Male)	Age 18–30 (vs. ≥ 55) yo	Age 31 - 54 (vs. ≥ 55) yo	Illness Yes (vs. No)	Medication Yes (vs. No)	Prior Infection Yes (vs. No)
Injection Site Pain	B (*SE*)	0.62 (0.27)	1.30 (0.43)	0.68 (0.38)	0.03 (0.43)	0.56 (0.42)	0.26 (0.41)
	AOR (*CI*)	1.86 (1.09–3.19)	3.68 (1.58–8.57)	1.97 (0.94–4.13)	1.03 (0.44–2.40)	1.75 (0.77–3.99)	1.30 (0.59–2.89)
	*Sig.*	0.023	0.002	0.075	0.950	0.182	0.518
Injection Site Swelling	B (*SE*)	0.16 (0.36)	2.20 (1.04)	1.61 (1.04)	0.00 (0.46)	0.23 (0.42)	0.41 (0.38)
	AOR (*CI*)	1.17 (0.58–2.39)	9.05 (1.17–70)	5.02 (0.66–38.38)	1.00 (0.41–2.47)	1.26 (0.55–2.90)	1.50 (0.71–3.17)
	*Sig.*	0.659	0.035	0.120	0.995	0.581	0.289
Injection Site Redness	B (*SE*)	0.28 (0.41)	1.94 (1.05)	1.43 (1.04)	–0.95 (0.50)	0.93 (0.41)	0.15 (0.44)
	AOR (*CI*)	1.33 (0.59–2.98)	6.93 (0.89–54.21)	4.18 (0.54–32.22)	0.39 (0.14 –1.03)	2.53 (1.12–5.68)	1.16 (0.49–2.75)
	*Sig.*	0.495	0.065	0.170	0.058	0.025	0.734
Local SideE ffects	B (*SE*)	0.68 (0.28)	1.31 (0.43)	0.76 (0.38)	–0.14 (0.44)	0.69 (0.44)	0.19 (0.41)
	AOR (*CI*)	1.97 (1.15–3.39)	3.72 (1.59–8.68)	2.14 (1.01–4.54)	0.873 (0.37–2.08)	2.00 (0.85–4.70)	1.21 (0.54–2.70)
	*Sig.*	0.014	0.002	0.047	0.759	0.113	0.641
Fatigue	B (*SE*)	0.86 (0.22)	1.87 (0.37)	1.10 (0.35)	–0.14 (0.29)	0.32 (0.28)	–0.15 (0.27)
	AOR (*CI*)	2.35 (1.52–3.64)	6.48 (3.13–13.38)	3.00 (1.52–5.92)	0.87 (0.49–1.55)	1.38 (0.80–2.39)	0.86 (0.51–1.46)
	*Sig.*	< 0.001	< 0.001	0.002	0.630	0.253	0.577
Headache	B (*SE*)	0.68 (0.24)	1.10 (0.40)	0.67 (0.38)	0.28 (0.30)	–0.29 (0.29)	–0.11 (0.28)
	AOR (*CI*)	1.97 (1.23–3.16)	3.00 (1.38–6.53)	1.96 (0.93–4.15)	1.32 (0.73–2.39)	0.75 (0.43–1.31)	0.90 (0.52–1.55)
	*Sig.*	0.005	0.006	0.079	0.354	0.305	0.692
Fever	B (*SE*)	0.27 (0.31)	1.31 (0.64)	0.87 (0.63)	0.42 (0.40)	–0.52 (0.39)	0.34 (0.33)
	AOR (*CI*)	1.31 (0.72–2.39)	3.70 (1.06–12.89)	2.38 (0.70–8.15)	1.53 (0.69–3.37)	0.59 (0.27–1.28)	1.41 (0.73–2.70)
	*Sig.*	0.374	0.040	0.166	0.294	0.183	0.302
Chills	B (*SE*)	0.42 (0.25)	0.83 (0.45)	0.53 (0.44)	0.09 (0.34)	–0.60 (0.32)	0.61 (0.28)
	AOR (*CI*)	1.52 (0.92–2.50)	2.29 (0.95–5.52)	1.70 (0.72–4.00)	1.10 (0.57–2.14)	0.55 (0.29–1.03)	1.85 (1.08–3.17)
	*Sig.*	0.099	0.066	0.227	0.783	0.063	0.026
Muscle Pain	B (*SE*)	0.31 (0.24)	0.90 (0.43)	0.74 (0.42)	0.63 (0.32)	–0.50 (0.31)	0.39 (0.28)
	AOR (*CI*)	1.36 (0.84–2.19)	2.45 (1.05–5.69)	2.10 (0.93–4.74)	1.88 (1.01–3.53)	0.61 (0.33–1.11)	1.48 (0.86–2.55)
	*Sig.*	0.209	0.038	0.074	0.048	0.107	0.153
Joint Pain	B (*SE*)	0.65 (0.32)	0.44 (0.47)	0.38 (0.45)	–0.18 (0.36)	0.54 (0.33)	0.40 (0.32)
	AOR (*CI*)	1.91 (1.02–3.60)	1.56 (0.62–3.91)	1.47 (0.61–3.52)	0.83 (0.42–1.67)	1.71 (0.90–3.27)	1.49 (0.80–2.78)
	*Sig.*	0.045	0.347	0.390	0.606	0.102	0.207
Nausea	B (*SE*)	0.32 (0.39)	–0.02 (0.68)	0.62 (0.63)	0.31 (0.51)	–0.79 (0.51)	–0.15 (0.46)
	AOR (*CI*)	1.38 (0.64–2.95)	0.98 (0.26–3.73)	1.86 (0.54–6.44)	1.37 (0.51–3.70)	0.46 (0.17–1.23)	0.86 (0.35–2.13)
	*Sig.*	0.411	0.973	0.329	0.537	0.120	0.742
Malaise	B (*SE*)	0.32 (0.28)	1.55 (0.56)	0.90 (0.55)	0.53 (0.36)	–0.39 (0.35)	0.41 (0.30)
	AOR (*CI*)	1.38 (0.80–2.37)	4.71 (1.57–14.11)	2.46 (0.84–7.24)	1.70 (0.85–3.42)	0.68 (0.35–1.34)	1.51 (0.84–2.73)
	*Sig.*	0.247	0.006	0.103	0.136	0.263	0.170
Lymphadenopathy	B (*SE*)	0.66 (0.50)	18.93 (5481)	18.99 (5481)	0.61 (0.50)	0.29 (0.48)	0.56 (0.43)
	AOR (*CI*)	1.94 (0.73–5.16)	16.64 × 10^6^	17.71 × 10^6^	1.85 (0.70–4.89)	1.34 (0.52–3.46)	1.75 (0.76–4.03)
	*Sig.*	0.183	1.00	1.00	0.217	0.544	0.193
Systemic Side Effects	B (*SE*)	1.07 (0.23)	1.86 (0.36)	1.26 (0.33)	–0.08 (0.33)	0.147 (0.31)	0.04 (0.31)
	AOR (*CI*)	2.90 (1.86–4.54)	6.40 (3.14–13.02)	3.54 (1.86–6.74)	0.93 (0.49–1.75)	1.16 (0.63–2.14)	1.04 (0.57–1.89)
	*Sig.*	< 0.001	< 0.001	< 0.001	0.810	0.638	0.896
General Side Effects	B (*SE*)	0.83 (0.34)	1.37 (0.49)	1.08 (0.43)	–0.01 (0.53)	0.09 (0.51)	1.07 (0.74)
	AOR (*CI*)	2.28 (1.18–4.44)	3.94 (1.51–10.29)	2.93 (1.27–6.77)	0.99 (0.36–2.78)	1.10 (0.40–2.98)	2.92 (0.68–12.54)
	*Sig.*	0.015	0.005	0.012	0.990	0.855	0.149
Oral Side Effects	B (*SE*)	0.60 (0.43)	1.34 (1.06)	2.02 (1.03)	0.13 (0.47)	–0.19 (0.45)	–0.71 (0.54)
	AOR (*CI*)	1.83 (0.79–4.22)	3.82 (0.48–30.72)	7.56 (1.00–57.08)	1.14 (0.45–2.87)	0.83 (0.34–2.01)	0.49 (0.17–1.43)
	*Sig.*	0.158	0.208	0.050	0.780	0.679	0.191
Skin-related Side Effects	B (*SE*)	0.87 (0.76)	17.11 (5509)	17.92 (5509)	–0.32 (0.85)	–0.29 (0.76)	0.52 (0.59)
	AOR (*CI*)	2.38 (0.53–10.63)	26.90 × 10^6^	60.71 × 10^6^	0.73 (0.14–3.85)	0.75 (0.17–3.34)	1.68 (0.53–5.32)
	*Sig.*	0.256	1.00	1.00	0.707	0.703	0.376

Binary logistic regression was used with a significance level (*Sig.*) of < 0.05. B = regression coefficient; *SE* = standard error; AOR = adjusted odds ratio; *CI* = confidence level of 95%.

## Data Availability

Data is contained within the article.
